# Combined therapy of positive interventions and cognitive training for reducing neurobehavioral symptoms of traumatic brain injury: A clinical case

**DOI:** 10.1192/j.eurpsy.2021.1340

**Published:** 2021-08-13

**Authors:** O. Khaustova, D. Assonov

**Affiliations:** Medical Psychology, Psychosomatic Medicine & Psychotherapy, Bogomolets National Medical University, Kiyv, Ukraine

**Keywords:** positive interventions, combined therapy, traumatic brain injury, cognitive training

## Abstract

**Introduction:**

There is a need to study therapies that may contribute to the successful rehabilitation of veterans with traumatic brain injury (TBI) and increase their effective interaction with the stressful environment, reduce the severity of symptoms. Combined short-term therapies may have potential.

**Objectives:**

To analyze the clinical case of combined psychological treatment of TBI in a Ukrainian combat veteran with reduced resilience

**Methods:**

The clinical case of Ukrainian combat veteran with TBI is presented. Montreal Cognitive Assessment (MoCA) was used to assess cognitive domains. Neurobehavioral symptom inventory (NSI) was used to assess neurobehavioral symptoms of TBI. CD-RISC was used to assess resilience. In addition to pharmacotherapy, the patient agreed to undergo a combined program of psychological therapy of 3 short-term positive intervention sessions and 3 cognitive training sessions.

**Results:**

MoCA result prior to treatment was 24 p., NSI – 38 p., CD-RISC – 44 p. (lower than in population). After the combined therapy, the results of the assessment with MoCA were 26 points, NSI was 17 points, CD-RISC – 47 points. Subjectively, the patient noted an improvement in emotional state, better resilience, and a significant reduction in the intensity of cognitive symptoms.

**Conclusions:**

Combining positive interventions with cognitive training can have the potential to significantly improve the neurobehavioral and cognitive functioning of war veterans with traumatic brain injury, and also possibly increase resilience. Further research in this direction will be conducted to obtain more reliable results.

